# A Chrysanthemum Heat Shock Protein Confers Tolerance to Abiotic Stress

**DOI:** 10.3390/ijms15035063

**Published:** 2014-03-21

**Authors:** Aiping Song, Xirong Zhu, Fadi Chen, Haishun Gao, Jiafu Jiang, Sumei Chen

**Affiliations:** 1 College of Horticulture, Nanjing Agricultural University, Nanjing 210095, China; E-Mails: aiping_song@aliyun.com (A.S.); zhuxirong88@126.com (X.Z.); chenfd@njau.edu.cn (F.C.); gaohs2002@163.com (H.G.); jiangjiafu@njau.edu.cn (J.J.); 2 Jiangsu Province Engineering Lab for Modern Facility Agriculture Technology & Equipment, Nanjing 210095, China

**Keywords:** abiotic stress tolerance, heat shock protein, transformation

## Abstract

Heat shock proteins are associated with protection against various abiotic stresses. Here, the isolation of a chrysanthemum cDNA belonging to the *HSP70* family is reported. The cDNA, designated *CgHSP70*, encodes a 647-residue polypeptide, of estimated molecular mass 70.90 kDa and pI 5.12. A sub-cellular localization assay indicated that the cDNA product is deposited in the cytoplasm and nucleus. The performance of *Arabidopsis thaliana* plants constitutively expressing *CgHSP70* demonstrated that the gene enhances tolerance to heat, drought and salinity. When *CgHSP70* was stably over-expressed in chrysanthemum, the plants showed an increased peroxidase (POD) activity, higher proline content and inhibited malondialdehyde (MDA) content. After heat stress, drought or salinity the transgenic plants were better able to recover, demonstrating CgHSP70 positive effect.

## 1. Introduction

Heat shock proteins (HSPs) are produced by both eubacteria and eukaryotes. They are typically induced by heat stress, but some are also triggered by other stresses, such as low temperature, drought, salinity, heavy metals and exogenously applied phytohormones [1]. HSPs are thought to function as chaperones involved in protein folding, assembly, translocation and degradation, and also help to stabilize proteins and membranes [2]. The most well-characterized HSPs belong to the *HSP70* family, which all share both a 44 kDa *N*-terminal ATP-binding [3] and a 25–30 kDa *C*-terminal substrate-binding region [4,5]. They serve a variety of functions, but are particularly associated with acquired thermotolerance [6]. The transgenic up-regulation of *HSP70* has been shown to enhance tolerance to high temperature [7,8], drought [9], and salinity [10]. Transgenic tobacco plants over-expressing *NtHSP70* are better equipped to survive heat/drought stress than those in which *NtHSP70* is expressed either normally or is suppressed [11]. The heterologous expression in *Arabidopsis thaliana* of a fungal (*Trichoderma harzianum*) *HSP70* provides some protection against damage caused by a variety of abiotic stresses [12].

Chrysanthemum (*Chrysanthemum morifolium*) is a leading ornamental species, which is rather susceptible to heat, drought and salinity stress. Here, we describe the isolation of an *HSP70* cDNA from the variety “Zhongshanzigui”, and show that that its heterologous expression in *A. thaliana* has a positive effect on stress tolerance. Furthermore, when constitutively expressed in the cut chrysanthemum variety “Jinba”, levels of tolerance to heat, drought and salinity were all improved.

## 2. Results and Discussion

### 2.1. The CgHSP70 Sequence

**Figure 1.**
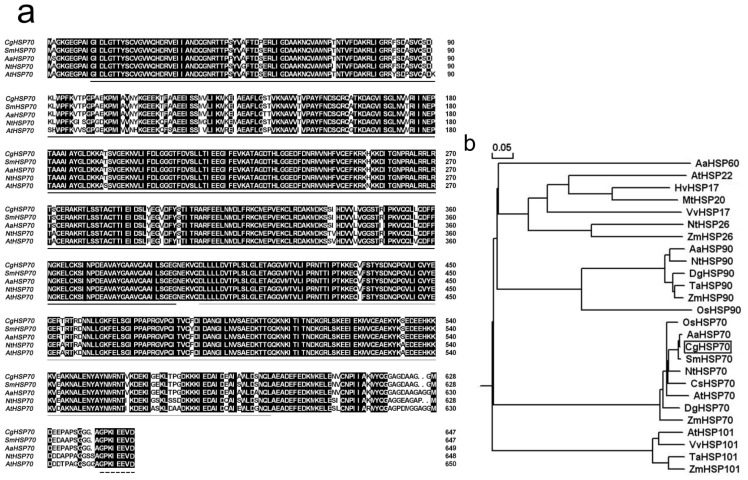
(**a**) Alignment of the deduced peptide sequences of CgHSP70 and those of related HSPs. Continuous dark line: ATPase domain, continuous grey line: peptide-binding domain, broken line: *C*-terminal signature motifs for organelle localization; (**b**) Phylogeny of CgHSP70 and related HSPs. Bootstrap values indicate the separation between adjacent branches and the scale bar represents 0.05 substitutions per site. The polypeptides along with their GenBank accession numbers are: AaHSP60 (ABX76300), AaHSP90 (ABX76302), AaHSP70 (ABX76301), AtHSP22 (NP_192763.1), AtHSP101 (AAF26423), AtHSP70 (NP_195870), HvHSP17 (CAA69172), MtHSP20 (ABD32352), VvHSP17 (ACZ48684), VvHSP101 (AAX08108), ZmHSP26 (NP_001105583), ZmHSP90 (ACO35045), ZmHSP70 (CAA27330), ZmHSP101 (AAD26530), NtHSP26 (BAA29065), NtHSP90 (AAS79798), NtHSP70 (AAP04522), DgHSP90 (ACX37414), DgHSP70 (ACD45076), TaHSP90 (ADF31771), TaHSP101 (AAF01280), OsHSP90 (BAA90487), OsHSP70 (CAA47948), CgHSP70 (AB503697), SmHSP70 (AAV97978), CsHSP70 (CAB72130). CgHSP70 highlighted by a black box.

The full length of the *CgHSP70* (AB503697) cDNA was 2205 bp, including a 22 bp polyA tail. The cDNA encodes a 647-residue polypeptide of estimated relative molecular mass (M_r_) 70.90 kDa and a isoelectric point (pI) of 5.12. The product includes the expected cytosolic compartmentalization sequence (GPKIEEVD) at its *C*-terminus [13,14], and its ATP binding domain and peptide-binding domain sequences are both strongly conserved; however its *C*-terminal sequence is rather divergent (Figure 1a). CgHSP70 showed high consensus to that of other plant HSP70 (Figure 1b). The highest levels of identity at the peptide level between CgHSP70 and other plant HSP70s were 98.9% with a gene from *Saussurea medusa*, 98.8% with a gene from *Ageratina adenophora*, 93.3% with a tobacco gene and 91.2% with an *A. thaliana* gene (Figure 1b). The expression analysis reveals that the *CgHSP70* gene is not heat responsive.

### 2.2. Sub-Cellular Localization of CgHSP70

**Figure 2.**
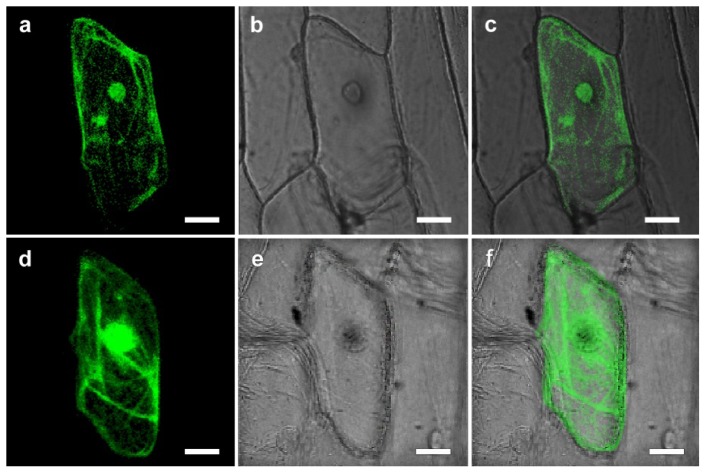
Sub-cellular localization of CgHSP70 in transiently transformed onion epidermal cells. (**a**–**c**) Onion epidermal cells transiently expressing *35S::CgHSP70-GFP*; (**d**–**f**) Onion epidermal cells transformed with a control construct (*35S::GFP*); (**a**,**d**) Dark field images to capture GFP fluorescence; (**b**,**e**) bright field images to capture cell features; (**c**,**f**) merged images. Scale bar: 25 μm.

Although CgHSP70 was predicted to be a cytosolic protein, it also, according to WoLF PSORT software [15], contains sequences associated with nuclear targeting. Experimentally, 35S::CgHSP70-GFP signals were detected in both the cytoplasm and the nucleus (Figure 2a–c), while the control transgene (*35S::GFP*) was expressed throughout the whole cell (Figure 2d–f). Members of the HSP70 family localize variously to the cytosol, nucleus, endoplasmic reticulum, mitochondria and chloroplasts [16]. The CgHSP70 sequence is highly similar to those of SmHSP70, AaHSP70, NtHSP70 and AtHSP70, all of which contain well conserved nuclear localization signals (Figure 1) [17]. Experimentally, CgHSP70 localized to the cytoplasm and nucleus (Figure 2). Several reports demonstrated that HSP70 would be translocated into the nucleus upon stress [18,19], moreover, HSP70 should be re-located from cytoplasm to nucleus upon heat stress [20]. Guinez *et al.* suggested that the nuclear transport of cytosolic proteins (HSP70) could be linked to *O*-GlcNAc glycosylation, but the mechanism involved remains unclear [21]. In the present study, CgHSP70 localization in the nucleus is very concentrated (Figure 2a–c), which suggested that the HSP is probably actively imported into the nucleus.

### 2.3. Stress Tolerance of A. thaliana Plants Heterologously Expressing CgHSP70

**Figure 3.**
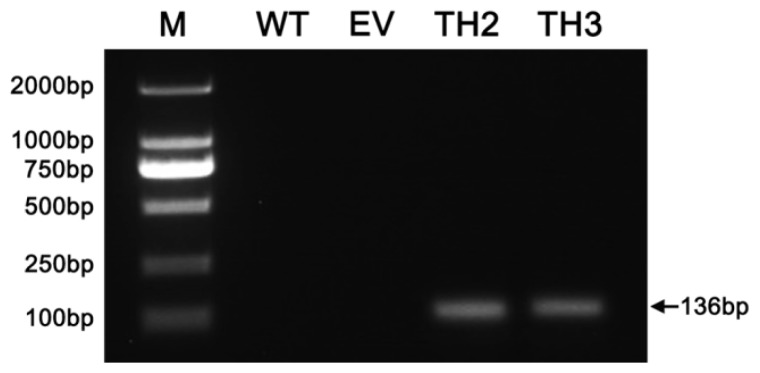
Transcription of *CgHSP70* in transgenic plants and non-transgenic *A. thaliana*.

**Figure 4.**
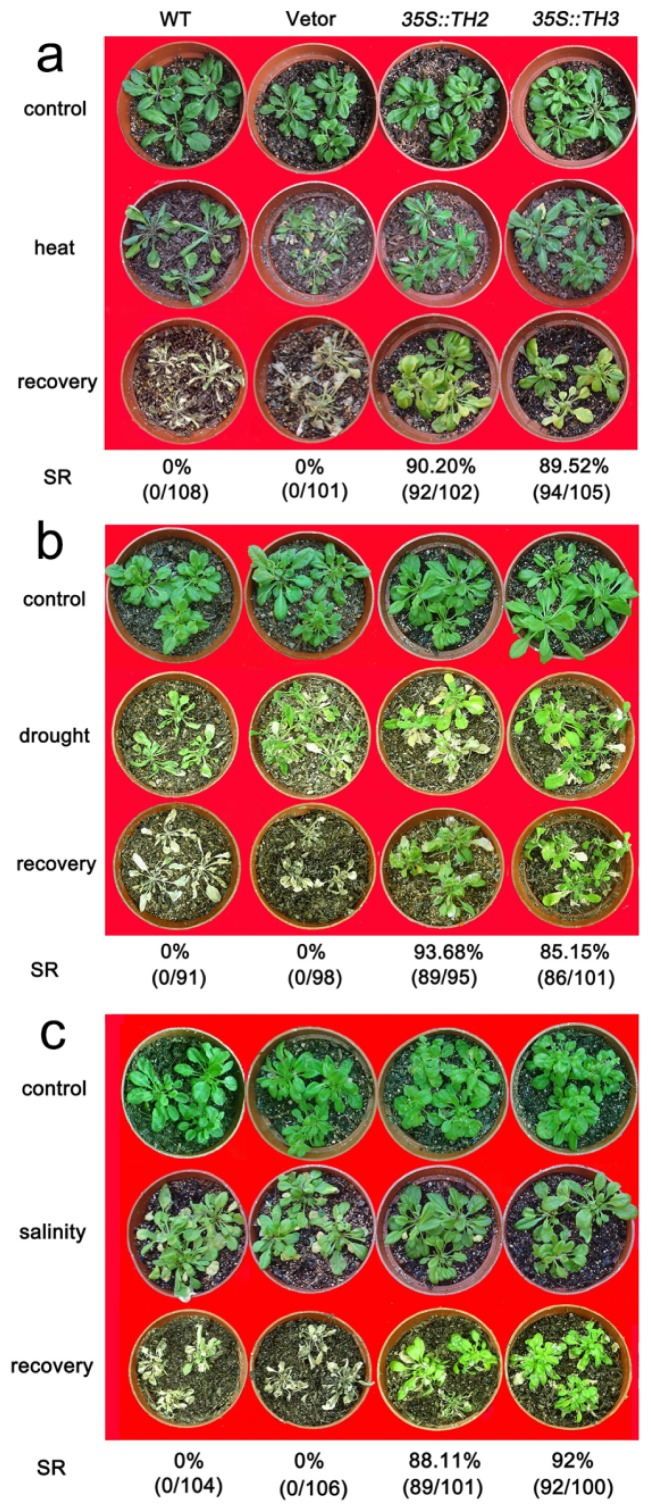
Stress tolerance of *A. thaliana* plants expressing *CgHSP70*. (**a**) The appearance of plants after exposure to 45 °C for 24 h and their survival rate recorded after a one week recovery period; (**b**) The appearance of plants after exposure to drought stress for 24 days and their survival rate recorded after a one week recovery period; (**c**) The appearance of plants after exposure to salinity stress for 15 days, and their survival rate recorded after a one week recovery period.

*CgHSP70* transcription was detected in lines 35S:TH2 and 35S:TH3, but not in either wild type (WT) or empty vector (EV) plants (Figure 3). In response to the heat stress, the leaves of WT and EV quickly became wilted, while the TH lines were less affected. The transgenic seedlings were better able to survive the heat stress, since 100% of the WT and EV seedlings died, whereas 90.2% of the 35S:TH2 plants and 89.5% of the 35S:TH3 plants recovered (Figure 4a). The transformants also showed an improved level of drought tolerance (Figure 4b); their respective survival rates (93.7% and 85.2%) are contrasted with the 100% death rate of WT and EV plants. Salinity stress did not completely inhibit the growth of the *CgHSP70* transformants, whereas it did cause the WT and EV plants to wilt. None of the WT and EV plants survived, whereas 88.1% and 92.0%, respectively, of the two transgenic lines did (Figure 4c).

The heterologous expression of various plant *HSP70* genes has been repeatedly shown to be correlated with an enhanced level of stress tolerance; For example, the antisense expression of *Nthsp70-1* in *A. thaliana* resulted in a reduction in the host’s thermotolerance [6]. Similarly, the introduction of an HSP70 cloned from a halotolerant cyanobacterium improved the thermotolerance of tobacco during both germination and early seedling growth [7]. When the rice mitochondrial *HSP70* gene (*mtHsp70*) was constitutively expressed in rice suspension culture cells, heat stress proved less potent in inducing apoptosis [22]. The heterologous expression of the *Porphyra seriata* mitochondrial gene *PsHSP70b* in *Chlamydomonas* improved survival and cell growth under heat stress conditions [23]. Drought stress tolerance proved also to be enhanced when *NtHSP70-1* was over-expressed [24]. The tolerance to stress, whether imposed by high temperature, drought or salinity, of *A. thaliana* was markedly improved when *CgHSP70* was constitutively expressed.

### 2.4. Transgene Transcription in Chrysanthemum

**Figure 5.**
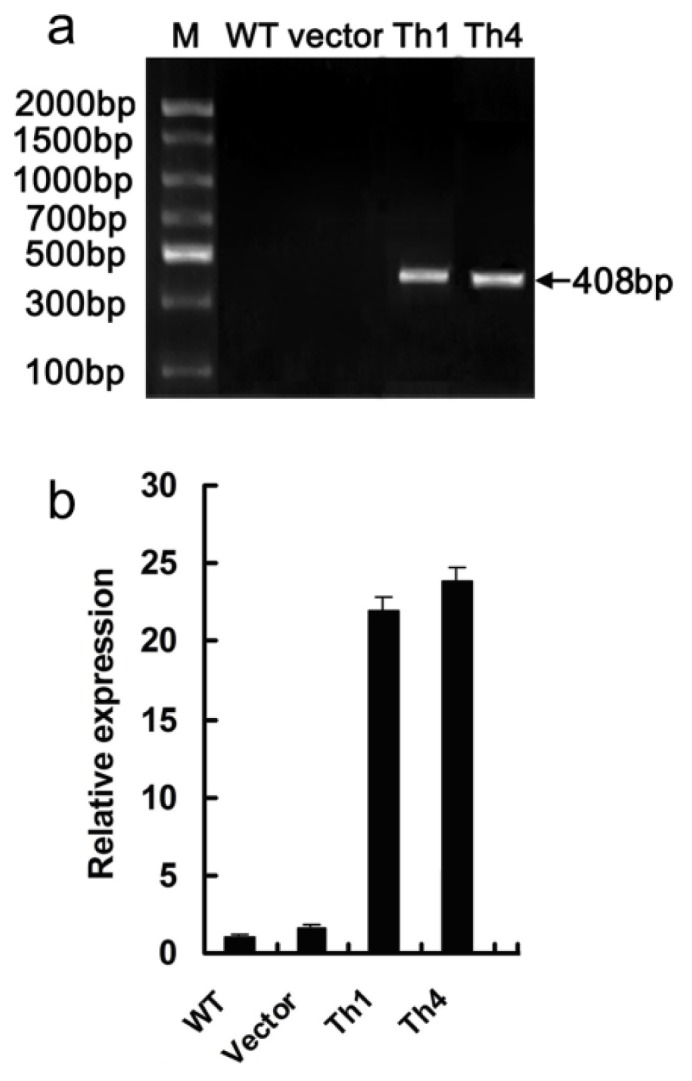
Chrysanthemum transformed with *CgHSP70*. (**a**) PCR assay for the presence of *hptII* in presumptive transgenic chrysanthemum regenerants. M: molecular size marker; (**b**) *CgHSP70* transcript abundance as measured by RT-qPCR.

Based on the presence of the *hptII* gene as detected by PCR, regenerants tolerating hygromycin were shown to be true transformants (Figure 5a). RT-qPCR analysis showed that *CgHSP70* transcript abundance in Th1 and Th4 was significantly higher than in either WT or EV plants (Figure 5b).

### 2.5. The Constitutive Expression of CgHSP70 Enhances the Abiotic Stress Tolerance of Chrysanthemum

**Figure 6.**
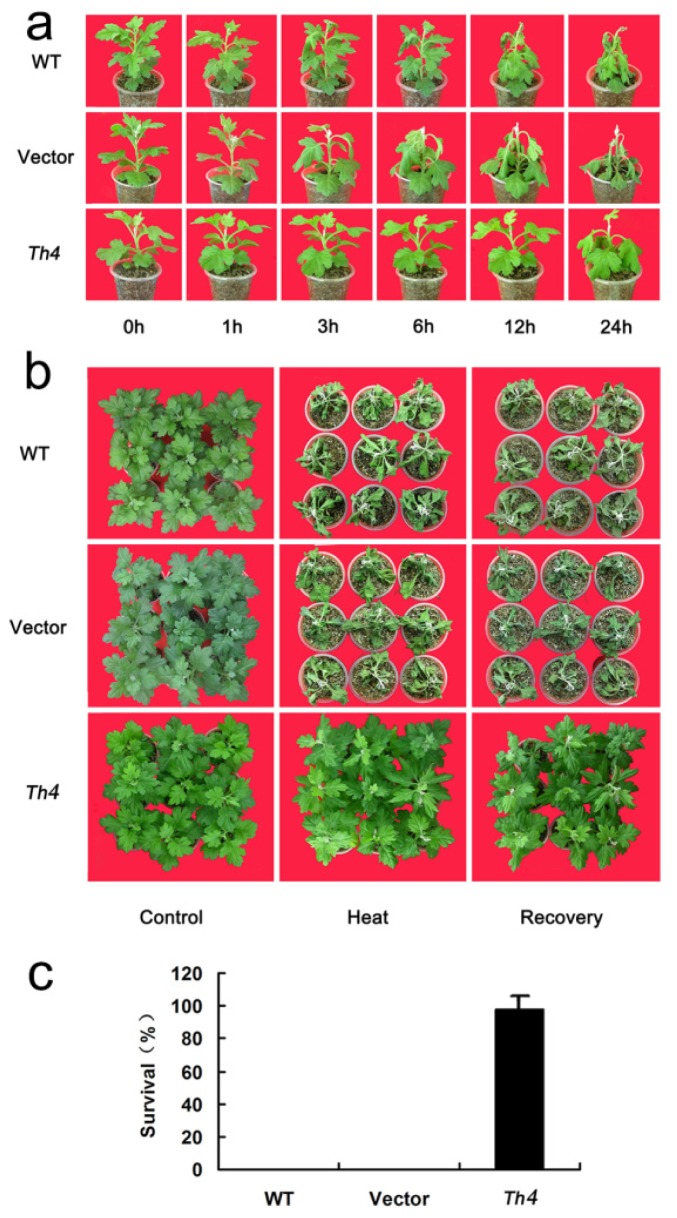
The heat tolerance of transgenic chrysanthemum. (**a**) The appearance of plants (WT, EV and Th4) after exposure to 45 °C for 0–24 h; (**b**) The appearance of plants (WT, EV and Th4) during the recovery phase; (**c**) The survival rate of transgenic and non-transgenic plants exposed to one week of high temperature. Error bars calculated from three replicates.

The semi-lethal temperatures (*LT*_50_) of Th1 and Th4 plants were significantly higher than those of either WT or EV plants (Table 1). Since Th1 and Th4 behaved very similarly, subsequent tests focused only on Th4. Heat stress caused the leaves of WT and EV plants to begin wilting within 3 h of the imposition of stress, and by 24 h they appeared to be extensively wilted; in contrast, Th4 plants showed no signs of wilting until 12 h after the imposition of stress, although by 24 h they were also heavily affected (Figure 6a). During the recovery stage, leaf damage occurred earlier and was more serious in the WT and EV plants (Figure 6b). Survival after a ten day recovery period was zero for WT and EV plants, but 98.1% for Th4 (Figure 6c).

**Figure 7.**
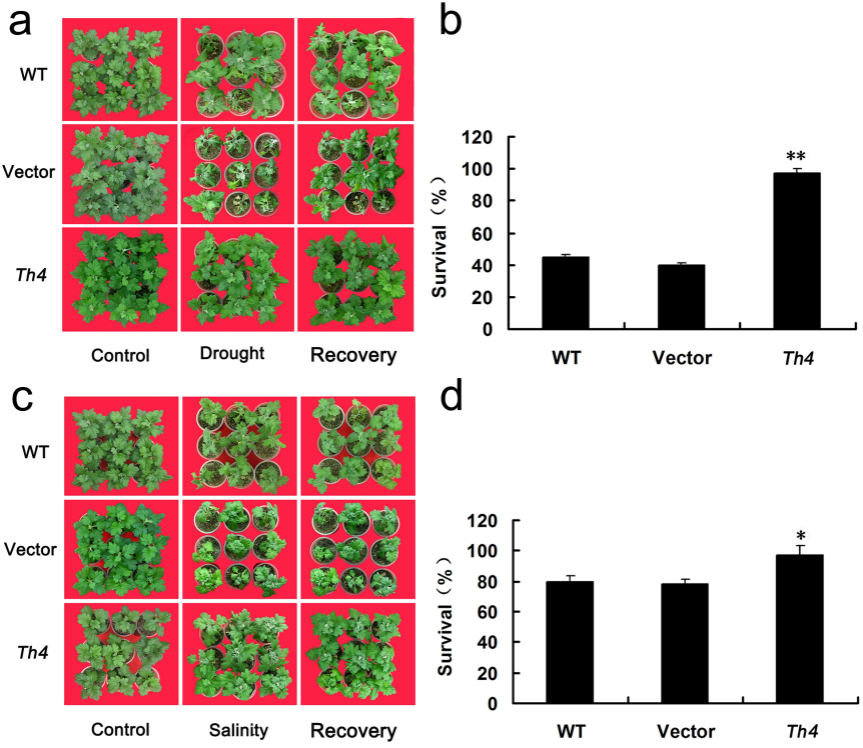
The drought and salinity tolerance of transgenic chrysanthemum. (**a**) The appearance of WT, EV and Th4 plants subjected to a 24 days period of no watering, and during the recovery period; (**b**) The survival rate of WT, EV and Th4 after a two-week recovery period. Error bars based on three replicates. Mean performances differing significantly from the WT and EV performance, as derived from a Tukey’s test, indicated by asterisks (** *p* < 0.01); (**c**) The appearance of WT, EV and Th4 plants subjected to salinity stress and during the recovery period; (**d**) The survival rate of WT, EV and Th4 after a two-week recovery period. Error bars are based on three replicates. Mean performances differing significantly from the WT and EV performance, as derived from a Tukey’s test, indicated by asterisks (* *p* < 0.05).

In response to drought stress, the damage symptoms (wilting, chlorosis, necrosis and death) was more severe for WT and EV than for Th4 plants (Figure 7a). The survival rate of WT and EV plants post recovery was, respectively, 45% and 40%, whereas it was 100% for Th4 (Figure 7b). As the concentration of salt was raised, WT and EV plants became increasingly wilted (Figure 7c). The rate of WT and EV plants post-treatment survival was, respectively 75% and 80% (Figure 7d). Th4 plants experienced only slight wilting in the lower part of the plant, and their post-treatment survival was 100%.

The transgenic improvement of chrysanthemum’s tolerance to abiotic stress has been the goal of a number of researchers. Thus, for example, Chen *et al.* showed that the over-expression of *CgDREBa* (which encodes a DREB transcription factor) has a positive effect on drought and salinity tolerance [25], while a similar experiment using the *A. thaliana* gene *AtDREB1A* conferred heat tolerance [26]. The constitutive expression of the transcription factor *CdICE1* also succeeded in enhancing the level of tolerance to low temperature, salinity and drought [27]. Here, the over-expression of *CgHSP70* markedly increased heat tolerance, which provides support to the suggestion made by Hong *et al.* [26]. It also had a positive effect on chrysanthemum’s drought tolerance as well as salinity tolerance.

### 2.6. Physiological Changes in CgHSP70 Over-Expression Chrysanthemum in Response to Abiotic Stresses

**Figure 8.**
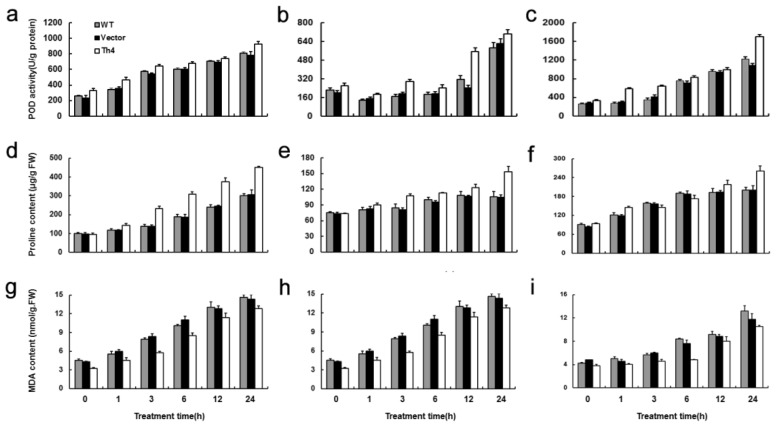
The ROS scavenging efficiency enhanced in the transgenic chrysanthemum. (**a**–**c**) leaf POD activity; (**d**–**f**) leaf proline content; (**g**–**i**) leaf MDA content during the treatment period. **a**, **d** and **g**: heat stress treatment; **b**, **e** and **f**: drought treatment; **g**, **h** and **i**: salinity treatment. Error bars are based on three replicates.

As is well known, heat, drought or salinity stresses would cause a marked increase in oxidative damage to plants [28–30]. High ROS levels are known to increase lipid peroxidation, allowing MDA concentration to be used as an indicator of oxidative stress peroxidation of membrane lipids [31]. An in principle straightforward means to achieve this is to promote the activity of enzymes involved in oxidative protection, such as peroxidase (POD), superoxide dismutase, ascorbate peroxidase and glutathione reductase, and to enhance the presence of anti-oxidant molecules, such as proline [32]. Here, POD activity increased after heat stress, drought and salinity treatments, but POD activity in Th4 was greater than that in WT and vector transformants at 24 h after treatments (Figure 8a–c). In addition, transgenic *CgHSP70* enhanced the presence of anti-oxidant molecules proline (Figure 8d–f), resulting in less membrane lipid peroxidation, displayed as less MDA content (Figure 8g–i). The results implied that reactive oxygen species (ROS)-induced damage to membranes in the *CgHSP70* over-expressors was less severe than that in the WT plants. The observations in the present study are consistent with previous reports in kidney cells, which showed that Hsp70 could regulate cellular redox status in response to ischemic stress, which may be important in Hsp70’s cytoprotective effects [33]. Chloroplasts are particularly vulnerable to ROS-induced damage [34], *Chlamydomonas reinhardtii HSP70B*, encoding a chloroplast-localized chaperone, may participate *in vivo* both in the molecular protection of the photosystem II reaction centers during photoinhibition and in the process of photosystem II repair [35]. It is suggested that *CgHSP70* over-expression may alleviate ROS damage to the chloroplasts. Collectively, overexpression of *CgHSP70* might reduce ROS and maintain secondary metabolism (higher proline content) in chrysanthemum transformants under heat, drought and salinity stress, which might partially contribute to less stress damage and higher survival rate, although other mechanisms were clearly also in play. Over-expression of *AtDREB1A* in chrysanthemum enhances tolerance to heat stress, where higher CO_2_ assimilation rates, Rubisco activity and sucrose-6-phosphate synthase (SPS) activity may contribute to the heat tolerance enhancement [26], inferring that photosynthetic gas-exchange assays should contribute to the understanding of mechanisms involved in the stress tolerance in *CgHSP70* over-expressed chrysanthemum, and will be a focus of our ongoing research.

## 3. Experimental Section

### 3.1. Plant Materials

The chrysanthemum varieties “Zhongshanzigui” and “Jinba” were both obtained from the Chrysanthemum Germplasm Resource Conservation Centre (Nanjing Agricultural University, Nanjing, China). Rooted cuttings were potted into a 1:1 (*v*/*v*) mixture of soil and vermiculite and grown in a greenhouse held at 23 ± 2 °C, 12 h photoperiod and 70% relative humidity (RH). *A. thaliana* ecotype Columbia plants were grown in a 1:1:1 (*v*/*v*/*v*) mixture of perlite, vermiculite and soilrite under a 16 h photoperiod (80–100 μm^−2^·s^−1^ illumination), with a day/night temperature of 23 °C/18 °C.

### 3.2. Isolation of CgHSP70

Total RNA was isolated from “Zhongshanzigui” leaves using the RNAiso reagent (TaKaRa, Tokyo, Japan), following the manufacturer’s instructions. The first cDNA strand was synthesized from 1 μg total RNA using SuperScript III reverse transcriptase (Invitrogen, Carlsbad, CA, USA), according to the manufacturer’s instructions. A fragment of the *CgHSP70* sequence was obtained by amplification of the cDNA library directed by a primer pair designed from the chrysanthemum EST AB503697 [36]. This was followed by 3′-RACE PCR, for which the primers used were HSP1, HSP2 and Adaptor-R (the sequences of these and the further primers mentioned below are given in Table 2); the resulting amplicon was gel-purified using a Biospin Gel Extraction kit (BioFlux, Hangzhou, China) and cloned into the pMD19-T easy (TaKaRa, Tokyo, Japan) vector for sequencing. Finally, the primer pair Q2-F/-R was designed to capture the putative 5′- and 3′-UTRs, and the resulting full-length cDNA was then cloned into pMD19-T easy for sequencing. The *CgHSP70* open reading frame (ORF) was amplified from this template using the primer pair HSP-ORF-F2/R2, which allowed for the inclusion of a *Sma*I and an *Xba*I cloning site.

### 3.3. Intracellular Localization of CgHSP70

The intracellular localization of the *CgHSP70* product was identified using a transient assay in onion epidermal cells. The *CgHSP70* ORF, lacking its stop codon, was amplified using a Phusion^®^ High-Fidelity PCR kit (New England Biolabs, Ipswich, MA, USA) with the primer pair CgHSP-Dra-F/-Not-R (Table 2), and the resulting amplicon cloned into pMD19-T for validation by sequencing. The same fragment was also inserted into the *Dra*I and *Not*I cloning sites of the pENTR™ 1A dual selection vector (Invitrogen, Carlsbad, CA, USA) using T4 DNA ligase (TaKaRa, Tokyo, Japan), and from there introduced into pEarleyGate 103 using LR Clonase™ II enzyme mix (Invitrogen, Carlsbad, CA, USA) [37]. The latter construct includes the expression marker gene *GFP*. Transient expression was induced biolistically and was monitored by detection of green fluorescent protein (GFP), using confocal laser microscopy [38].

### 3.4. Transformation of A. thaliana

The pCAMBIA1301 cassette, containing the CaMV-35S promoter, the *hptII* gene and the *nos* terminator was modified by substituting the *Sac*I restriction site for an *Xba*I one, using the primer sets 1301-F/-R1 and 1301-F/-R2 (Table 2). The *HSP70* ORF was cleaved by restriction with *Sma*I and *Xba*I and introduced into *Sma*I and *Xba*I digested pCAMBIA1301 to produce pCAMBIA1301-CgHSP70. Both pCAMBIA1301 and pCAMBIA1301-CgHSP70 were transformed into *Agrobacterium tumefaciens* EHA105 using the freezing transformation method. *A. thaliana* was transformed with either pCAMBIA1301 or pCAMBIA1301-CgHSP70 using the floral dip method [39], and putative transformants were selected by germination on Murashige and Skoog medium containing 50 μg/mL hygromycin. Positive selections were advanced to the T_2_ and T_3_ generations, with RT-PCR analysis, based on the primer pair HSP-F/-R, being applied to select true transformants. The wild type, transformants carrying an empty vector, transformants carrying pCAMBIA1301 and transformants carrying pCAMBIA1301-CgHSP70 are referred to hereafter as, respectively, “WT”, “EV”, “35S:TH2” and “35S:TH3”.

### 3.5. Stress Tolerance of Transgenic A. thaliana

A set of two-week old WT, EV, 35S:TH2 and 35S:TH3 plants were well watered and then exposed to a 24 h period at 45 °C under continuous illumination (~100 μmol·m^−2^·s^−1^). They were left to recover at 25 °C for one week before noting the survival rate and recording any changes to morphology. Drought tolerance was measured on a similar set of WT, EV, 35S:TH2 and 35S:TH3 plants by withholding water for 24 days; survival was recorded seven days after re-watering [38]. To assess salinity tolerance, an identical set of plants was exposed to 300 mM NaCl for five days after being sequentially watered with 100, 200 mM NaCl at five day intervals [40]. Plant survival was recorded after a one-week recovery period.

### 3.6. Transformation of Chrysanthemum

Leaf disks (0.5 cm diameter) taken young leaves of “Jinba” plants were used as explant. The pCAMBIA1301 and pCAMBIA1301-CgHSP70 cassettes were introduced separately using *A. tumefaciens* EHA105-mediated leaf disk transformation [41], and selection for transformants was achieved by including 10 μg/mL in the culture medium. Surviving plantlets were potted into a sterile mixture of 1:1 (*v*/*v*) soil and vermiculite and grown in a greenhouse. A PCR test based on the genomic DNA of these plants and targeting the *hptII* gene (primer pair HptII-F/-R, see Table 2) was used to confirm successful transformation. Individuals carrying pCAMBIA1301-CgHSP70 are referred to hereafter as “Th”, and those that carry the pCAMBIA1301 as “EV”. Real time quantitative PCR (RT-qPCR) was used to determine *HSP70* transcript abundance following Gu *et al.* [42]. Total RNA was extracted from the shoot tips of WT, EV and Th plants at the 6–10 leaf stage using the Trizol reagent (Invitrogen, Carlsbad, CA, USA), and treated with DNaseI (TaKaRa, Tokyo, Japan) to remove any contaminating DNA. cDNA was synthesized using Reverse Transcriptase M-MLV (RNase H^−^) (TaKaRa, Tokyo, Japan). The qRT-PCRs were run on a Bio-Rad iQ5 PCR platform (Hercules, CA, USA) using SYBR Green qPCR SuperMix Universal (Invitrogen, Carlsbad, CA, USA). The reference sequence was a fragment of the chrysanthemum *GAPDH* gene (DK941612) [42], assayed with primer pair GAPDH-F/-R, while *CgHSP70* was assayed with the primer pair HSP-F/-R (Table 2). Three replicates of each RT-qPCR were performed, and the data are presented in the form mean ± SE. Relative transcription levels were calculated by the 2^−ΔΔ^*^C^*^t^ method [43].

### 3.7. Evaluation of the Abiotic Stress Tolerance of Transgenic Chrysanthemum

Rooted cuttings of transgenic and non-transgenic chrysanthemum were potted into a 1:1 (*v*/*v*) mixture of soil and vermiculite and grown in a greenhouse (23 ± 2 °C, 12 h photoperiod, 70% RH) until they reached the 6–10 leaf stage. Each assay was based on 20 plants per line.

#### 3.7.1. Heat Tolerance

The plants were subjected to a 24 h exposure at 45 °C under continuous illumination (~100 μmol·m^−2^· s^−1^) and subsequently were allowed to recover for one week following description of Hong *et al.* [26]. The plants were photographed before the heat stress, and then after 1, 3, 6, 12 and 24 h. In addition, the semi-lethal high temperature (*LT*_50_) parameter, as used to quantify low temperature tolerance [27], was obtained as follows: 0.1 g of chopped leaf (0.5 cm^2^ pieces) was placed in 20 mL deionized water and heated to either 40, 45, 50, 55 or 60 °C for 20 min (four replicates per temperature). The electrical conductivity (EC) of water was measured to generate a relative conductivity (REC%) value, being the ratio of the EC measured before heating and after heating. The REC% was fitted to the logistic function K/(1 + ae^−bx^), where x was the temperature and K the saturation capacity of cell damage rate calculated using Rcpsys [44]. The *LT*_50_ was given by ln[(1/a)]/b.

#### 3.7.2. Drought Tolerance

The procedure followed that described by Hong *et al.* [45]. Water was withheld for nine days from plants at the 8–10 leaf stage. The growing environment was adjusted to 30 °C, 30% RH. After the stress period, the plants were kept fully watered for two weeks (23 ± 2 °C, 70% RH), and the survival rate was then counted.

#### 3.7.3. Salinity Tolerance

After withholding water for four days, the plants were subjected to 300 mM NaCl for five days after being sequentially watered with 100, 200 mM NaCl at five days intervals, respectively [40]. The plants were uprooted to rinse their roots, and were then replanted into fresh soil and left to recover for two weeks, after which their survival rate was recorded.

#### 3.7.4. Measurement of Physiological Indices

POD activity, MDA and proline content of transgenic plants under heat, drought and salinity stress were measured. POD activity was estimated from the absorbance change at 470 nm caused by the oxidation of guaiacol according to the method of He *et al*. [46]. One unit of POD activity was defined to be equivalent to the amount of enzyme required to degrade 0.01 μmol of substrate per min per mg protein. Protein concentration was measured based on the method of Bradford [47], using bovine serum albumin as a standard. Lipid peroxidation was measured in terms of malondialdehyde (MDA) concentration. MDA concentration was determined using the thiobarbituric acid method according to Yin *et al.* [48]. The level of lipid peroxidation was expressed as nmol·g^−1^ fresh weight. Proline concentration was determined by the ninhydrin method following Guha *et al.* [49] with minor modifications. A 0.5 g leaf sample was homogenized in 10 mL 3% (*w*/*v*) aqueous sulfosalicylic acid, and the homogenate was centrifuged at 12,000× *g* for 10 min. The supernatant was boiled in 3 mL acid ninhydrin for 30 min, cooling to room temperature. The absorbance of the supernatant was recorded at 520 nm. Proline concentration was expressed as μg·g^−1^ fresh weight.

### 3.8. Statistical Analysis

The data were analysed using SPSS v17.0 software (SPSS Inc., Chicago, IL, USA). The percent data were angular transformed before conducting an analysis of variance, then Tukey’s test was used to identify means differing significantly from one another.

## 4. Conclusions

The heterologous expression of an *HSP70* cDNA from chrysanthemum variety “Zhongshanzigui” in *A. thaliana* has a positive effect on stress tolerance. Furthermore, when constitutively expressed in the cut chrysanthemum variety “Jinba”, levels of tolerance to heat, drought and salinity were all improved.

## Figures and Tables

**Table 1.** semi-lethal temperature (*LT*_50_) of transgenic plants and non-transgenic plants.

Plant lines	Semi-lethal temperature *LT*_50_ (°C)
WT	47.7 ± 02 ^A^
*Vector*	47.9 ± 0.6 ^A^
*Th1*	50.2 ± 0.2 ^B^
*Th4*	50.3 ± 0.3 ^B^

Values with different superscripts indicate significant differences at *p* < 0.01 by Tukey’s test. Values represent mean ± S.E.

**Table 2.** The PCR primers used in this study.

Primer	Sequences (5′-3′)	Annotation
HSP1	TCAGTCCAAAGCGACATCAAA	For 3′-RACE
HSP2	CAGCCTACTTCAACGACTCAC	For 3′-RACE
Adaptor-R	AGCAGTGGTATCAACGCAGAG	Universal primers for 3′-RACE
Q2-F	CACAAACCCTAATTCTTTTTCATACA	Full length cloning
Q2-R	AGTAGAACAGATAAATATCGACCACA	Full length cloning
HSP-ORF-F2	CcccgggATGGCTGGTAAAGGGTGAA	Open reading frame (ORF) cloning
HSP-ORF-R2	GCtctagaGTCGACCTCTTCGATCTTGG	Open reading frame (ORF) cloning
CgHSP-Dra-F	AGGCtttaaaATGGCTGGTAAAGGTGA	Intracellular localisation of CgHSP70
CgHSP-Not-R	GTgcggccgcGAGTCGACCTCT	Intracellular localisation of CgHSP70
1301-F	TCCcccgggGTTATGACGCTGGGAATGTTTT	pCAMBIA1301 vector modified
1301-R1	GCtctagaAGATAATGCCACAGCACCTCTT	pCAMBIA1301 vector modified
1301-R2	cGAGCTCtctagaAGATAATGCCACAGCACCTCTT	pCAMBIA1301 vector modified
GAPDH-F	GCTGTATCCCCATTCGTT	qRT-PCR
GAPDH-R	AGAAGGCAAGCTCAAGGG	qRT-PCR
HSP-F	GCTTGCTGAGGCTGATGAGT	qRT-PCR
HSP-R	ACCTGATGGTGCGGGTTCCTC	qRT-PCR
HptII-F	CTCGATGAG CTGATGCTTTGGG	verifying positive transformants
HptII-R	GCTTCTGCGGGCGATTTGTGTA	Verifying positive transformants
